# Regional socio-environmental characteristics associated with inadequate prenatal care during pregnancy: an ecological study of 47 prefectures in Japan

**DOI:** 10.1186/s12884-021-04100-0

**Published:** 2021-09-13

**Authors:** Eri Osawa, Tomoko Kodama

**Affiliations:** Department of International Health and Collaboration, National Institute of Public Health, 2-3-6 Minami Wako-shi, Saitama, 351-0197 Japan

**Keywords:** Prenatal care, Ecological study, Regional socio-environmental factors, Universal health coverage, Japan

## Abstract

**Background:**

Prenatal care (PNC) is a crucial health service that reduces the potential risks of adverse pregnancy and childbirth outcomes. It is monitored as one of the indicators of Universal Health Coverage (UHC) under the United Nations’ Sustainable Development Goals. However, there are still mothers who do not use PNC, even when UHC has been achieved. As there have been few reports on the impact of local socio-environmental characteristics within the country, this study aimed to examine the association between local socio-environmental factors and inadequate use of PNC in Japan.

**Methods:**

We conducted an ecological analysis of 47 prefectures in Japan using public open data. The dependent variables were the inadequate use of PNC, which are the rates of pregnant women who missed visiting PNC until 28 weeks’ gestational age (GA) or those who never attended PNC before childbirth, and the independent variables were prefectural data of socio-economic, educational, and healthcare workforce-related factors. Multiple logistic regression analysis was used to examine the associations.

**Results:**

The rate of pregnant women with late PNC initiation and never attending PNC before childbirth was 3.00–11.24 and 0.23–8.06 per 1000 pregnant women, respectively. Population numbers and densities, divorce rates, percentages of non-Japanese nationalities, and low percentages of high school enrolment were positively associated with inadequate PNC use. There was no statistically significant association with healthcare workforce, such as the number of obstetricians and gynaecologists.

**Conclusions:**

This ecological study revealed that inadequate PNC use is more common in urban areas with more non-Japanese nationality and lower education enrolment. There may be a need to provide education for those who do not have access to reproductive health education, such as that offered in high schools. Further studies are required to examine factors that affect access to PNC in Japan.

## Background

Prenatal care (PNC) has been recognised as a means of monitoring maternal and foetal health, and it leads to a safe and healthy pregnancy, safe delivery, and a healthy newborn [1]. The World Health Organization recommends at least eight contacts for antenatal care during pregnancy from the first trimester to delivery [2]. Universal health coverage (UHC): financial risk protection, access to quality essential healthcare services, and access to safe, effective, quality, and affordable essential medicines and vaccines are global targets of the United Nations’ Sustainable Development Goals. One of the UHC indicators being monitored worldwide is access to PNC [3].

Inadequate PNC use is reported to be a risk factor for poor pregnancy and birth outcomes [4–7]. A study that included approximately 28 million deliveries over an 8-year period in the United States defined inadequate care as the initiation of PNC after 4 months of gestation or attending fewer than half of the expected visits [4]. The results showed that the inadequate PNC group had a higher risk of preterm birth, small for gestational age (SGA), stillbirth, and early and late neonatal mortality. A population-based retrospective cohort study conducted in Canada also showed that inadequate or no PNC use was associated with a higher risk of low birth weight and SGA (20 and 30%, respectively) [5].

Community-level socioeconomic and social environments have also been associated with inadequate PNC use [8, 9]. A previous study in the United States defined late entry into PNC as no initiation of care until 7 months of gestation or not at all and showed that residing in a poor area is associated with the likelihood of beginning care late, even after adjusting for individual-level variables, such as age, education, and race [8]. The highest rate of inadequate PNC use, defined by the number of PNC visits and initiation time, was among women living in areas with the lowest average family income and highest proportion of unemployment, who are recent immigrants, and from single-parent families and families with low education levels [9]. In Japan, maternity health check-ups are recommended once every 4 weeks from the beginning of pregnancy to 23 weeks of gestation, once every 2 weeks from 24 to 35 weeks of gestation, and once per week from 36 weeks of gestation to childbirth. Over the entire pregnancy, at least 14 check-ups are subsidised at the public expense [10]. Although in principle, every pregnant woman has access to PNC in Japan, some women experience childbirth without any care, including PNC. A previous study in Japan found severe and complicated socioeconomic conditions to be common among cases with no PNC [11]. Further, one 12-year hospital-based study in Japan showed that 3.2% of pregnant non-Japanese residents experienced childbirth without PNC and that the mothers’ background indicated social isolation and financial hardship due to no health insurance or partner [12]. However, due to the lack of systematically recorded data on socioeconomic status and residence in the prenatal health surveillance system in Japan, no study has examined access to PNC at a regional level and the association between regional socio-environmental characteristics and late and no PNC use. It is useful to understand the regional situation and assess the community in order to consider tailored interventions for maternal care programs to the regional characteristics. Therefore, in the present study, we examined the relationship between regional socio-environmental factors and the rates of late and no PNC use among pregnant women in the prefectures of Japan.

## Methods

### Study design

This was an observational ecological study using open public data in Japan. We calculated the rate of late initiation of PNC and never attending PNC in Japan’s 47 prefectures and analysed the associated prefectural-level factors.

### Indicators and operational definitions

The rate of inadequate PNC use in each prefecture was obtained from Pregnancy Reports that the prefectures submit annually to the Ministry of Health, Labour and Welfare (MHLW) as part of the Report on Regional Public Health Services and Health Promotion Services [13]. In Japan, when pregnancy is diagnosed by doctors or midwives, the woman reports her pregnancy to a residential municipality [14] and then receives vouchers for health check-ups during pregnancy with a maternal and child health handbook. Every June, each municipality submits the annual pregnancy report numbers, which are divided into five groups based on GA (i.e., < 11 weeks, 12–19 weeks, 20–27 weeks, 28 weeks to childbirth, and after childbirth) to the MHLW through the prefecture. We classified the reports submitted at ≥28 weeks GA (including those submitted after childbirth) as late PNC initiation and those submitted after childbirth as never attending PNC. Cases per 1000 pregnant women within 5 years, from 2014 to 2018, were calculated for the respective rates.

The explanatory variables were calculated using different data sources. The total population, population density, percentage of non-Japanese nationality, and percentage of single-parent households were calculated using figures from the 2015 population census [15]. The divorce rate (per 1000 people) was calculated using the mean figure among annual vital statistics of 5 years, from 2015 to 2019 [16]. Prefectural income per capita—an economic indicator—was the mean of the most recent 5 years (2013–2017) of prefectural economic statistics calculated from the gross product in a prefecture and the income of the prefecture’s residents [17]. We also used the Gini coefficient as an indicator of economic disparity within a prefecture, referencing 2014 figure from the National Consumption Survey, a national sampling survey targeting 30,000 households to investigate household consumption [18]. The results of the School Basic Survey, which collects data, such as school attendance and teachers’ employment status from kindergartens to universities [19], were used as educational indicators. Variables analysed included the percentage of high school graduates advancing to university or vocational school, percentage of middle school graduates advancing to high school, and proportion (per 1000 students) of long-term absentees (≥30 days missed per school year) in middle school due to refusal to attend school. The variables were reported as 5-year means from 2013 to 2017, the latest reported year. The healthcare indicator was the number of obstetricians and gynaecologists (OBGYNs) per 10,000 women aged 15–49 years and was calculated using age-based population statistics on women from the 2015 population census [14] and figures from the 2016 Statistics of Physicians, Dentists and Pharmacists, which compiles numbers based on a biennial self-report [20].

### Statistical analysis

First, the distribution of the rates of women with late PNC initiation, never attending PNC visits before childbirth, and explanatory variables were calculated.

Next, to examine the relationships between the two outcomes and explanatory variables, we calculated the crude odds ratio (OR) for all variables. Variables that were statistically associated with either the rate of late PNC initiation or never attending PNC before childbirth by single logistic regression were entered into the multiple logistic regression model. Furthermore, single-parent family and income per capita which were associated with inadequate use of PNC in the previous empirical studies [8, 9], were also entered into the model. All analyses were conducted using the STATA MP statistical package, version 16 (StataCorp LP, College Station, TX, USA). For this analysis, the two outcome variables were divided by the mean, and the explanatory variables were divided into 2–4 categories. In the analysis, a two-tailed test was used, and *p* < 0.05 was considered statistically significant.

## Results

Table 1 describes the rates of inadequate PNC use and the demographic, socioeconomic, educational and healthcare workforce indicators in the prefectures. The mean rate of late initiation of PNC (per 1000 pregnant women) was 5.44 (standard deviation [SD], 1.83; range, 3.00–11.24). The mean rate of never attending PNC visits (per 1000 pregnant women) was 1.42 (SD, 1.38; min, 0.23; max, 8.06). Socioeconomic indicators, such as the mean population (× 10^− 4^) was 270.4 (SD, 272.9; range 57.3–1351.5), and the mean percentage of non-Japanese nationals in the population was 0.99% (SD, 0.58; range, 0.26–2.80). The mean percentage of single-parent family was 9.28% (SD, 0.89; range, 7.53–12.66), and the mean divorce rate (per 1000 people) was 1.66 (SD, 0.20; range, 1.31–2.52). The mean annual prefectural income per capita was 2.85 million yen (SD, 0.49; range, 2.19–5.44). The mean Gini coefficient was 0.31 (SD, 0.01; range 0.28–0.34). For educational indicators, the mean percentage of high school graduates advancing to university or vocational school and percentage of middle school graduates advancing to high school were 50.94% (SD, 6.56; range, 39.18–66.08) and 97.02 (SD, 1.17; range, 93.2–98.88), respectively. The mean number of OBGYNs (per 10,000 women aged 15–49 years) was 4.58 (SD, 0.69; range, 2.90–6.05).

**Table 1:** The rates of inadequate PNC use and descriptions of socioeconomic indicators in 47 prefectures

Variable	Mean	SD	Min	Max
Rates of inadequate PNC use				
Pregnant women who initiated PNC at 28 weeks GA or later (per 1000 pregnant women)				
	5.44	1.83	3.00	11.24
Pregnant women who never attended PNC visits before childbirth (per 1000 pregnant women)				
	1.42	1.38	0.23	8.06
Socioeconomic indicators				
Population (×10^−4^)	270.4	272.9	57.3	1351.5
Population density (persons/m^3^)	655.3	1194.4	68.6	6168.7
Non-Japanese nationality population (%)	0.99	0.58	0.26	2.80
Single-parent families (%)	9.28	0.89	7.53	12.66
Divorce rate (per 1000 population)	1.66	0.20	1.31	2.52
Annual prefectural income per capita (million yen)				
	2.85	0.49	2.19	5.44
Gini coefficient	0.31	0.01	0.28	0.34
Educational indicators				
Percentage of enrolment into advanced educational institutions among high school graduates (%)				
	50.94	6.56	39.18	66.08
Percentage of enrolment into high school among middle school graduates (%)				
	97.02	1.17	93.2	98.88
Rate of long-term absence due to refusal to attend school (per 1000 students)				
	28.37	3.50	20.76	37.11
Healthcare workforce indicators				
Number of OBGYNs (per 10,000 women aged 15–49)				
	4.58	0.69	2.90	6.05

*SD* Standard Deviation

*GA* Gestational Age

*PNC* Prenatal Care

*OBGYNs* Obstetricians and Gynaecologists

Table 2 shows the association between inadequate PNC use and each explanatory variable using logistic regression analysis. In the simple logistic regression on late PNC initiation, for the total population, with the smallest group (< 1 million) as the reference, the crude OR of the largest group (≥5 million) was 16.0 (95% confidential interval [CI]: 1.32–194.62]. Population density was not associated with outcome. Concerning the percentage of non-Japanese nationals in the population, the OR of the high-percentage group (more than the mean) was 4.28 (95% CI: 1.21–15.15). Regarding the divorce rate, the OR of the high-rate group (more than the mean) was 7.0 (95% CI: 1.65–29.69). Neither economic indicator was statistically significant. No educational indicator was associated with the rate of late PNC initiation. In the adjusted model for the rate of late PNC initiation, high divorce rate (more than the mean) was still statistically significant.

**Table 2:** Correlations between inadequate PNC use and socioeconomic, educational, and healthcare indicators in univariate logistic regression analysis

	N	PNC initiation at 28 weeks GA or later						Never attending PNC before childbirth					
		crude OR	95% CI		a_OR	95% CI		Crude OR	95% CI		a_OR	95% CI	
Socioeconomic indicators													
Total population (×10^−4^)													
under 100	9	Reference						Reference					
100–199	21	4.00	0.41	38.65				1.33	0.12	14.87			
200–499	8	4.80	0.38	59.89				1.14	0.06	21.87			
500 or more	9	16.00*	1.32	194.62				10.00	0.85	117.02			
Population density (persons/m^3^)													
under 300	25	Reference			Reference			Reference			Reference		
300–999	15	1.29	0.32	5.13	0.55	0.06	4.96	1.13	0.17	7.67	0.15	0.01	2.85
1000 or over	7	6.43	1.00	41.20	1.17	0.07	18.19	18.33*	2.39	140.39	2.05	0.11	38.27
Non-Japanese nationality population (%)													
less than mean (≤0.9941)	27	Reference			Reference			Reference			Reference		
more than mean (> 0.9941)	20	4.28*	1.21	15.15	14.71	0.89	242.96	4.31	0.95	19.53	1.69	0.12	24.64
Single-parent families (%)													
less than the mean (≤9.284436)	26	Reference			Reference			Reference			Reference		
more than the mean (> 9.284436)	21	0.80	0.24	2.66	2.09	0.19	23.37	0.45	0.10	2.02	0.31	0.03	3.64
Divorce rate (per thousand)													
less than the mean (≤1.668244)	21	Reference			Reference			Reference			Reference		
more than the mean (> 1.668244)	26	7.00*	1.65	29.69	7.22*	1.21	43.11	10.59*	1.22	92.25	6.15	0.45	84.26
Annual prefectural income per capita (million yen)													
under 2.5	10	Reference			Reference			Reference			Reference		
2.5 or over and under 3.0	24	0.96	0.19	4.82	0.38	0.04	3.47	1.80	0.18	18.47	0.25	0.01	6.43
3.0 and over	13	2.72	0.48	15.47	0.28	0.01	6.16	5.63	0.54	58.91	0.37	0.01	18.01
Gini coefficient													
less than the mean (≤0.3064681)	26	Reference						Reference					
more than the mean (> 0.3064681)	21	1.69	0.51	5.60				1.31	0.32	5.32			
Educational indicators													
Percentage of enrolment into advanced educational institutions among high school graduates (%)													
less than the mean (≤50.94128)	23	Reference						Reference					
more than the mean (> 50.94128)	24	1.13	0.34	3.70				1.58	0.38	6.55			
Percentage of enrolment into a high school among middle school graduates (%)													
less than the mean (≤97.01957)	21	Reference			Reference			Reference			Reference		
more than the mean (> 97.01957)	26	0.41	0.12	1.37	1.20	0.17	8.75	0.14*	0.02	0.73	0.15	0.01	1.84
Rate of long-term absence due to refusal to school (per 1000 students)													
less than the mean (≤28.36566)	24	Reference						Reference					
more than the mean (> 28.36566)	23	0.89	0.27	2.93				1.76	0.43	7.31			
Healthcare workforce indicators													
No. of OBGYNs (per 10,000 women aged 15–49 years)													
less than the mean (≤4.583474)	24	Reference						Reference					
more than the mean (> 4.583474)	23	0.61	0.18	2.04				0.63	0.15	2.61			

*p < 0.05

Two outcome variables were divided by the mean, and the explanatory variables were divided into 2–4 categories

*GA* Gestational Age

*PNC* Prenatal Care

*OR* Odds Ratio

*a-OR* adjusted Odds Ratio

*CI* Confidential Interval

*OBGYNs* Obstetricians and Gynaecologists

In the logistic analysis, the rate of never attending PNC before childbirth was associated with large population density (> 1000), high divorce rates (more than the mean), and high percentage of enrolment into high school (more than the mean) (crude OR [95% CI]: 18.33 [2.39–140.39], 10.59 [1.22–92.25], and 0.14[0.02–0.73], respectively). In the adjusted model, no variables were statistically significant.

## Discussion

In this study, we showed that the current rate of inadequate PNC use in Japan is related to access to PNC. As a result, some prefectures have a very high rate of inadequate PNC use. Although all pregnant women have access to PNC through public subsidies, there is a disparity in the level of access to PNC among prefectures. We also examined the association between the rate of late initiation and never attending PNC visits before childbirth, along with regional-level socioeconomic and educational indicators using regional Japanese data. To the best of our knowledge, this is the first ecological study to investigate the association between access to PNC and regional socio-environmental factors in Japan.

In the present study, we found that the rates of inadequate PNC use were high in prefectures with large populations or high population densities, such as prefectures with large urban areas. Living in urban areas has the advantage of access to social determinants of health, such as education, employment, and housing. However, there is the possibility of unequal access and social exclusion from these factors [21]. As such, urban areas offer improved access to healthcare services while simultaneously making access difficult for some people. Regarding adverse childbirth outcomes, a previous study conducted in urban cities in the Netherlands reported that the highest risks were observed in deprived neighbourhoods within cities [22]. A similar phenomenon might have led to the present study results.

In addition, inadequate PNC use appeared to be higher in prefectures with higher divorce rates. In a survey in Osaka Prefecture, Japan, childbirth without care during pregnancy was found in a wide range of age groups, from teenagers to women in their 40s, with unmarried people accounting for approximately 70% of the total population [11]. This indicates that the rate of late initiation and never attending PNC before childbirth seems to be increased in areas where more people have unstable marital status.

Prefectures with a higher proportion of non-Japanese residents tended to have higher rates of late PNC initiation. Posthumus et al. found that ethnic minority, as an individual-level factor, and ethnic minority density, as a neighbourhood-level factor, were both associated with a higher risk of late initiation of care, with PNC commencing after 14 weeks of gestation [23]. Using a different definition of PNC use as an outcome measure, Heaman et al. also reported a higher risk of inadequate PNC use in women living in neighbourhoods with a high number of immigrants [9].

Regarding educational indicators, the rate of never attending PNC visits tended to be lower in the group with a high enrolment rate in high school among middle school graduates; however, it was also associated with the rate of late PNC initiation. Heaman et al. reported that areas with < 9 years of education have a high percentage of women receiving inadequate PNC [9]. This tendency was also observed in our study. It is possible that a strengthened access to reproductive health education to the young may improve PNC use.

While previous research observed a higher risk of inadequate PNC use in areas with low-income households [24], in the present study, income and income disparity within a prefecture were not associated with the rate of inadequate PNC use. However, the previous study analysed the impact of community environment on inadequate PNC use by postal codes, while our study used prefectures. Postal codes have smaller areas compared with prefectures, the smallest of which has a population of approximately 570,000. Thus, the aggregated data at the prefectural level of the economy may not represent individual economic indicators.

This study is the first attempt to calculate the rates of late and no PNC use using official Japanese administrative data. It also examined regional socio-environmental factors for rates of late and no PNC use using Japanese public data. However, this study has several limitations. First, the PNC use indicator in this study was based on when the local government received the pregnancy report. Therefore, our data may not accurately represent the actual PNC utilization. Second, we may have missed pregnant women who give birth without Pregnant Report because we analysed data from the Report. Third, we used data by prefecture using aggregate data that may have a small impact on pregnant women’s behaviour towards attending PNC. Finally, because we examined regional correlations, we did not include data on the personal backgrounds of pregnant women and did not consider the association between regional and individual factors in our analyses. However, because all the data used came from official statistics, data and data collection methods were highly reliable.

## Conclusion

Regional disparities in the rate of late PNC initiation and the rate of never attending PNC visits before childbirth were observed at the prefectural level in Japan. Among the social, economic, educational, and healthcare indicators, rates of late and no PNC use were positively correlated with population numbers, population densities, divorce rates, and percentage of non-Japanese residents and were negatively correlated with the percentage of enrolment into high school among middle school graduates.

Japan currently has no mechanism to comprehensively examine the individual factors for pregnant women, period of PNC initiation, number of PNC visits/contacts before delivery, and delivery status. In future, further analyses of the factors that determine the conditions surrounding pregnancy and delivery, access to care, and delivery outcomes by way of increased data use and linkage are required to improve access to PNC in order to achieve UHC, and the state where no pregnant woman gets left behind, in Japan.

## Data Availability

The web pages from which all data generated or analyzed in this study were obtained are presented in the references section.

## Abbreviations

PNC: Prenatal Care
UHC: Universal Health Coverage
SGA: Small for Gestational Age
GA: Gestational Age
MHLW: Ministry of Health, Labour and Welfare
OBGYN: Obstetricians and Gynaecologist
OR: Odds Ratio
SD: Standard Deviation
CI: Confidential Interval
